# Dermoscopy of Syringotropic and Folliculotropic Mycosis Fungoides

**DOI:** 10.5826/dpc.1004a69

**Published:** 2020-10-26

**Authors:** Ružica Jurakić Tončić, Jaka Radoš, Danijela Ćurković, Ivana Ilić, Stefano Caccavale, Mirna Bradamante

**Affiliations:** 1 University Department of Dermatology and Venereology, University Hospital Centre and School of Medicine, Zagreb, Croatia; 2 University Hospital Centre Zagreb, Department of Pathology and Cytology, University of Applied Health Sciences, Zagreb, Croatia; 3 Department of Dermatology, Second University of Naples, Italy

**Keywords:** dermoscopy, primary cutaneous lymphoma, mycosis fungoides, syringotropic mycosis fungoides, folliculotropic mycosis fungoides

## Introduction

Syringotropic mycosis fungoides (STMF) is a rare variant of mycosis fungoides (MF) with prominent involvement of eccrine structures. It typically presents with red/skin-colored/brown papules, patches, scaly plaques, nodules, and lichenification, along with pruritus, hair loss, and poor heat tolerance and sweating [1]. Whether STMF is a subtype of folliculotropic MF (FMF) or a separate entity is still a matter of debate [1]. Most STMF patients have a benign, indolent course and a better prognosis compared to patients with FMF [1]. Histopathology is the gold standard for diagnosis of MF and STMF.

## Case Presentation

**Figure 1:**
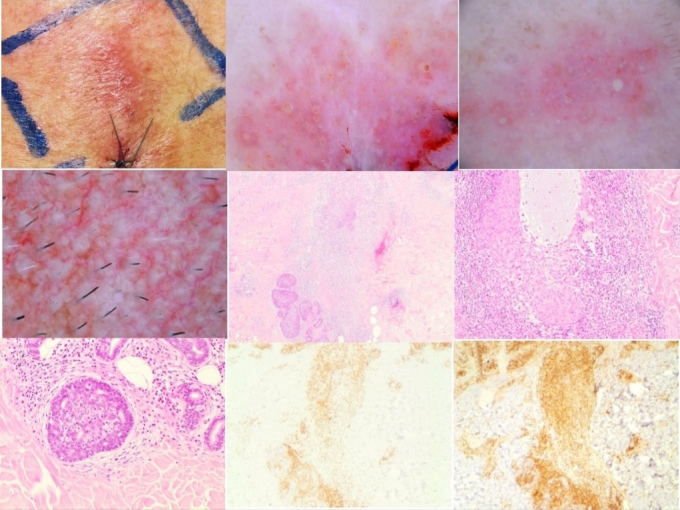
Patient 1. Clinical and dermoscopic presentation: dermoscopic images were taken from the lesion on which biopsy was done. The lesion was on the brachial region of the patient. Along with typical dermoscopic presentation of MF on other parts of the body, on selected body parts, lesions presented with follicular accentuation and with follicular plugging. Follicular accentuation and plugging were more visible when higher magnification was used. Clinical staging of the patients at the time of evaluation was T2N0M0B0. (A) Clinical presentation of lesion from where the biopsy was taken. (B–C) Dermoscopy of the lesion from where the biopsy was taken. (D) Dermoscopy of the skin from the presternal area. (E–H) Histologic presentation of the lesion from where the biopsy was done. (E) Follicular and ecrrine gland involvement by dense infiltrate of atypical lymphocytes (H&E, ×4). (F) Marked folliculotropism, peri- and intrafolllicular lymphoid infiltration with mucin deposition (H&E, ×10). (G) Syringometaplastic structures of eccrine glands surrounded and infiltrated by atypical lymphocytes (H&E, ×20). (H) Immunohistochemical staining was positive for T lymphocytes (CD4; ×4) (I) Lymphocytic infiltrate was composed of CD3+ lymphocytes (×4). Clinical photography was taken with a Canon Powershot SX520 HS and the dermoscopic image with a Nikon/DermLite Photo.

We present 2 patients with a diagnosis of STMF, both with co-expression of CD4+/CD8+. Patient 1, a 54-year-old man, presented with erythematous patches and plaques on the neck, face, arms, and back accompanied with pruritus. Diagnosis was made using full-thickness skin biopsy; the sites were chosen upon dermoscopic examination of the most prominent lesions. Few papules and milia-like cysts were seen. Other lesions appeared as classical MF both clinically and dermoscopically (poorly elevated plaques and dry and lichenificated skin). No alopecia, follicular hyperkeratosis, or mucinorrhoea were noted; palms and soles were not affected. A diagnosis of folliculotropic and syringotropic MF was made. Staging using the WHO/EORTC classification indicated stage IB (T2N0M0B0) (Figure 1).

**Figure 2:**
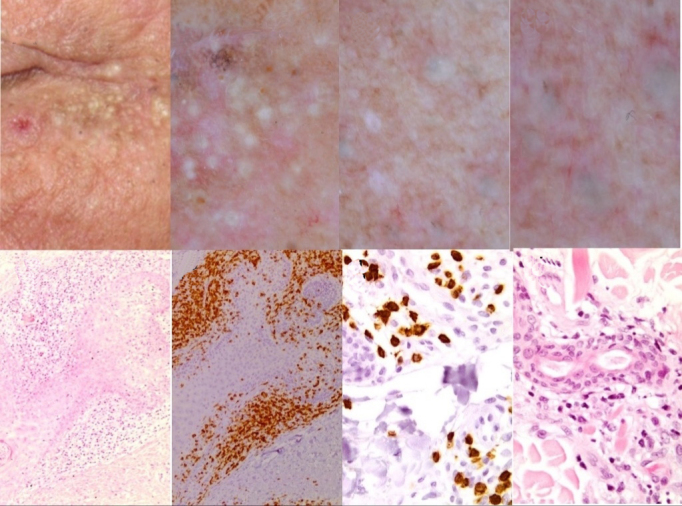
Patient No 2. Clinical and dermoscopic presentation of the lesion on the face. In this patient follicular accentuation is less visible, follicular obliteration and plugging are more pronounced, and bluish, not well-defined areas are seen Clinical staging of the patients at the time of evaluation was T4N2M0B0. (A) Clinical presentation of the lesion from where biopsy was taken. (B–D) Dermoscopic appearance of the lesion from where the biopsy was taken. Dermoscopy shows obliteration of the follicles and in some parts bluish, not well -defined structures where eccrine involvment was found. (E–H) Histology of the lesion from where biopsy was taken. (E) Atypical lymphocytes surround and invade follicular epithelium (H&E, ×10). (F) Atypical lymphocytes were positive for CD3+ on immunohistochemistry (×10). (G) CD3+ lymphocytes have a predilection for eccrine ducts (×20). (H) Infiltration of eccrine ducts with atypical small to medium-sized lymphocytes (H&E, ×20). Clinical photography was taken with a Canon Powershot SX520 HS and the dermoscopic image with a Nikon/DermLite Photo.

Patient 2, a 64-year-old female, presented with erythematous papules, plaques, comedones, and milia localized on the neck and chest (that later progressed to the trunk and extremities), accompanied with pruritus. Full-thickness skin biopsy was performed after the dermoscopic examination of the most prominent skin lesions, and a diagnosis of folliculotropic and syringotropic MF was established (stage IIIB), phenotype CD4+/CD8+, CD30−. Staging according to ISCL/EORTC classification was T4N2M0B0 (Figure 2).

Dermoscopy in both cases showed obliteration of the follicles, follicular accentuation, and follicular plugging (these observations for FMF have already been published), along with loss of terminal follicles, comedo-like openings, and interconnected regular-appearing structureless patches [1] (Figures 1 and 2). Orange color and follicular plugging were seen, corresponding to dense, diffuse, and perifollicular lymphocytic infiltrate. Similar observations were made in both patients, with one major distinction. We have never observed the bluish structures with unsharp borders before in FMF patients, as in the case of Patient 2, who presented with more advanced disease. These structures were not observed in pure FMF (Figure 2). There are probably 2 explanations for this observation. In the Patient 1, histology showed that follicles were more prominently affected and dermoscopic images correlated more with FMF, while in the Patient 2 eccrine glands were predominantly affected. It is interesting to note that this kind of bluish color is commonly observed with other eccrine type of tumors (such as eccrine hidrocystoma) [2]. Accumulation of pigmented substances in eccrine glands was excluded by the pathologist; therefore, the bluish color is not a result of this kind of pigmentation.

## Conclusions

Since currently there is no data on dermoscopic images of STMF, and these 2 patients differ in stage of the disease, we calculate that the differences observed are due to the stage of the disease and probably correspond to severity of the infiltration of eccrine ducts with atypical lymphocytes (staging of the patients). In order to understand this entity more, we need dermoscopic studies with a larger number of the patients in different stages of the disease.
